# The efficacy and safety of tigecycline for the treatment of bloodstream infections: a systematic review and meta-analysis

**DOI:** 10.1186/s12941-017-0199-8

**Published:** 2017-04-05

**Authors:** Jian Wang, Yaping Pan, Jilu Shen, Yuanhong Xu

**Affiliations:** Department of Clinical Laboratory, The First Affiliated Hospital of Anhui Medical University, Anhui Medical University, Hefei, 230022 Anhui China

## Abstract

Patients with bloodstream infections (BSI) are associated with high mortality rates. Due to tigecycline has shown excellent in vitro activity against most pathogens, tigecycline is selected as one of the candidate drugs for the treatment of multidrug-resistant organisms infections. The purpose of this study was to evaluate the effectiveness and safety of the use of tigecycline for the treatment of patients with BSI. The PubMed and Embase databases were systematically searched, to identify published studies, and we searched clinical trial registries to identify completed unpublished studies, the results of which were obtained through the manufacturer. The primary outcome was mortality, and the secondary outcomes were the rate of clinical cure and microbiological success. 24 controlled studies were included in this systematic review. All-cause mortality was lower with tigecycline than with control antibiotic agents, but the difference was not significant (OR 0.85, [95% confidence interval (CI) 0.31–2.33; P = 0.745]). Clinical cure was significantly higher with tigecycline groups (OR 1.76, [95% CI 1.26–2.45; P = 0.001]). Eradication efficiency did not differ between tigecycline and control regimens, but the sample size for these comparisons was small. Subgroup analyses showed good clinical cure result in bacteremia patients with CAP. Tigecycline monotherapy was associated with a OR of 2.73 (95% CI 1.53–4.87) for mortality compared with tigecycline combination therapy (6 studies; 250 patients), without heterogeneity. Five studies reporting on 398 patients with *Klebsiella pneumoniae* carbapenemase-producing *K. pneumoniae* BSI showed significantly lower mortality in the tigecycline arm than in the control arm. The combined treatment with tigecycline may be considered the optimal option for severely ill patients with BSI.

## Background

Bloodstream infections (BSI) are potentially life-threatening diseases. BSI was defined as at least 1 positive blood culture for a recognized pathogen and clinical symptoms consistent with bacteraemia. They can cause serious secondary infections, such as infective endocarditis and osteomyelitis, and may result in severe sepsis. Meanwhile, BSI due to multidrug-resistant (MDR) organisms has been associated with multiple poor outcomes, including increased length of hospital stay, health care costs and a high rate of morbidity and mortality.

Tigecycline is a glycylcycline with a broad spectrum of antibacterial activity. The emergence of MDR strains infections has been extensively observed worldwide and has become a priority issue over past decade. Tigecycline is a useful alternative to face the challenges of many MDR organisms. Tigecycline has a large volume of distribution of 7–10 l/kg [1], penetrating well into different tissues, it has been approved for the treatment of complicated skin and soft-structure infections (cSSSI), complicated intra-abdominal infections (cIAI), and community-acquired bacterial pneumonia (CAP). Tigecycline is not indicated for treatment of diabetic foot infection or for hospital-acquired or ventilator-associated pneumonia [2]. The use of tigecycline in bacteremia is controversial because of its low serum levels with standard dosing [3].

Attention should be paid by clinicians, because tigecycline was associated with higher mortality than comparator antibiotics [4–6]. However, a recent meta-analysis showed that the drug was not associated with significantly higher mortality than comparator antibiotics and was as effective as comparators when the analysis was restricted to patients who received tigecycline for approved indications [7]. A prospective study demonstrates that tigecycline plus prolonged infusion standard-dose imipenem/cilastatin, showed good clinical efficacy on VAP patients with XDR-Ab VAP bacteremia [8]. The increased mortality associated with tigecycline is not yet well understood in the treatment of BSI. Therefore, we systemically searched and analysed the current available evidence to assess clinical effectiveness of tigecycline for the treatment of BSI.

## Methods

### Literature search

Relevant studies were identified through PubMed, Embase and hand-searched from inception until October 2016.The search terms were:“(tigecycline OR TGC OR tygacil) and (bacteraemia OR bacteremia OR bloodstream infection OR sepsis OR septicaemia)”. No language restrictions were applied.

### Study selection

Any article providing the clinical outcomes of patients treated for bloodstream infections caused by any etiological agent was considered eligible for inclusion in the review. Prospective and retrospective observational cohort studies examining the association between tigecycline use (on hospital admission or previous users) and the outcomes of bacteremic patients were included. The outcome of interest was overall hospital mortality at the longest follow-up at each single study. Case reports and case series including fewer than 10 infected patients treated with tigecycline were excluded from the review.

### Data extraction

The extracted data consisted of the main characteristics of a study (first-author name, year of publication, country, study period, and design), main characteristics and underlying diseases of the study population, number of patients with infections BSI, the causative pathogen(s), sites of infections, and antibiotic treatment (combination therapy or monotherapy). Clinical outcomes (mortality, treatment failure) of patients in each treatment group were recorded as well.

### Statistical analysis

We chose mortality as the primary outcome, because of the high mortality rates among patients with BSI, while the secondary outcomes were: clinical response, microbiological response, adverse effects, and emergence of resistance. Microbiological response was defined as successful when eradication or sterile culture results were obtained during or after the antibiotic therapy. Because there are no standard criteria to assess clinical response and adverse events, we accepted the criteria as reported in each study.

All statistical analyses were performed using the comprehensive meta-analysis V2.2 (BioStat, Englewood, NJ). Among the controlled studies, the between-study heterogeneity was assessed using the I^2^ test, whereby I^2^ values >50% were defined as indicating heterogeneity. Either fixed-effects (Mantel–Haenszel method) or random-effects (DerSimonian and Laird method) models were used, depending on the heterogeneity result. If no heterogeneity was found, meta-analysis was done using the Mantel–Haenszel fixed-effects model. Binary outcomes from controlled studies were expressed as odds ratios (OR) with their 95% confidence intervals (CI), and continuous outcomes were expressed as the mean difference between 2 groups. Egger regression, as well as the Begg methods, was used to evaluate publication bias. All P values were two-tailed, and a P value of ≤0.05 was considered statistically significant. Some statistical analysis was performed by using the SPSS statistical software (version 19; SPSS Inc., Chicago, IL). Categorical variables were evaluated by using the χ^2^ test or 2-tailed Fisher’s exact test, as appropriate. Subgroup analyses for mortality and clinical cure were planned for bacteraemic patients. Comparisons were subcategorized by the type of infection. A funnel plot was used to assess small-study effects.

## Results

### Literature search results

1540 potential articles were identified; 56 case reports and clinical series including less than 10 infected patients were excluded; 41 duplicates and 18 single-arm studies were excluded; 22 studies were ruled out because they did not present clear treatment regimens or detailed clinical outcomes; 24 articles were excluded due to few patients in each group. Ultimately 24 studies met the inclusion criteria, 24 controlled studies (1961 patients) included in this systematic review.

### Study characteristics

The features of the 24 trials are described in Table 1. Five of them were prospective cohort studies, 7 were retrospective studies. All of the included controlled studies had an NOS score >3. Most patients in the included studies were critically ill, with most of them in ICU.

**Table 1:** Characteristics of included studies

Reference	Study years	Location	Type of study	Type of infection	Causative pathogen(s)	Mortality assessed	Sample size(no.of tigecycline/control patients)	Concomitant antibiotics administeredin tigecycline group	Compatator	Tigecycline dose	Control regimen dose
Oliva et al. [22]	Na	Multicenter	Prospective, double-blind phase 3	cIAI with bacteremia	Mix	Undetermined	14/27	None	Imipenem cilastatin	Initial dose of 100 mg, followed by 50 mg every 12 h	500 mg followed by 500 mg per 6 h combined imipenem and cilastatin
McGovern et al. [11]	2001–08	USA	Comparative studies, phase 3 and 4	Bacteremia	Mix	Overall	162/163	Na	Na	Na	Na
Daikos et al. [23]	2009–10	Greece	Retrospective	BSI	CP-Kp	At 28 days	94/81	Colistin aminoglycoside carbapenem	Colistin aminoglycoside carbapenem	For tigecycline the total daily dose was 100–200 mg administered in two divided dosages	1 g imipenem and meropenem every 8 h, 5 mg/kg gentamicin and amikacin once daily
Jean et al. [8]	2013	Taiwan	Prospective	VAP with bacteremia	XDR-Ab	At 14 days	28/56	Ipipenem/cilasta	Sulbactam + imipenem/cilastatin	Na	Na
Florescu et al. [24]	2003–05	Multicenter	Double-blind, phase 3	Bacteremia	MRSA VRE	At 12–37 days after the last dose	14/20	None	Vancomycin or linezolid	Initial dose of 100 mg, followed by 50 mg every 12 h	1 g per 12 h followed by 600 mg per 12 h vancomycin plus linezolid
Sacchidanand et al. [25]	Na	Multicenter	Randomized, double-blind, phase 3	cSSSI with bacteremia	XDR-Ab	Overall	8/22	None	Vancomycin plus aztreonam	Initial dose of 100 mg, followed by 50 mg every 12 h	1 g per 12 h followed by 2 g per 12 h vancomycin plus aztreonam
Liou et al. [26]	2007–13	Taiwan	Retrospective	Secondary bacteremia	Acinetobacter	At 14 days	17/65	Ampicillin-sulbactam sulbactam levofloxacin ceftazidime	Na	Standard dose of tigecycline	Na
Cheng et al. [27]	2010–13	Taiwan	Prospective	Bacteremia	XDR-Ab	At discharge	29/55	Colistin	Colistin carbapenem	Initial dose of 100 mg, followed by 50 mg every 12 h	2.5–5 mg/kg/d of colistin base divided over 8 or 12 h
Tanaseanu et al. [28]	2003–05	Multicenter	Randomized, double-blind, phase 3	CAP with bacteremia	*Streptococcus pneumoniae*	At 7–23 days after the last dose	22/40	None	Levofloxacin	Initial dose of 100 mg, followed by 50 mg every 12 h	500 mg every 24 h levofloxacin
Tumbarello et al. [29]	2010–11	Italy	Retrospective	BSI	KPC-Kp	At 30 days	70/48	Colistin gentamicin meropenem	Colistin gentamicin meropenem	Every 12 h (100–200 mg/day)	Every 8–12 h for a total daily dose of 6,000,000–9,000,000 IU colistin; 4–5 mg/kg every 24 h gentamicin; 2 g every 8 h meropenem
Zarkotou et al. [30]	2008–10	Greece	Observational	BSI	KPC-Kp	Overall	22/13	Colistin gentamicin carbapenem amikacin	Colistin gentamicin carbapenem	Na	Na
Bucaneve et al. [31]	2008–10	Multicenter	Prospective, open-label	Bacteremia	Mix	Overall	86/94	Piperacillin/tazobactam	Piperacillin/tazobactam	Initial dose of 100 mg, followed by 50 mg every 12 h	4.5 g piperacillin/tazobactam every 8 h
Papadimitriou-Olivgeris et al. [32]	Na	Greece	Single-centre observational study	BSI	KPC-Kp	At 30 days	27/9	Colistin gentamicin	Colistin gentamicin	Na	Na
Gardiner et al. [19]	Na	Multicenter	Retrospective, randomized, 7 double-blind and 1 open-label, phase 3	Bacteremia	Mix	Na	91/79	Na	Na	Na	Na
Breedt et al. [33]	2002–03	Multicenter	Randomized, double-blind, phase 3	cSSSI with bacteremia	Mix	Overall	15/10	None	Vancomycin-aztreonam	Initial dose of 100 mg, followed by 50 mg every 12 h	1 g vancomycin plus 2 g aztreonam per 12 h
Babinchak et al. [34]	2002–04	Multicenter	Randomized, double-blind, phase 3	cIAI with bacteremia	Mix	Overall	40/50	None	Imipenem-cilastatin	Initial dose of 100 mg, followed by 50 mg every 12 h	500 mg followed by 500 mg per 6 h combined imipenem and cilastatin
Ellis-Grosse et al. [35]	2001–04	Multicenter	Randomized, double-blind, phase 3	cSSSI with bacteremia	Mix	Overall	23/24	None	Vancomycin-aztreonam	Initial dose of 100 mg, followed by 50 mg every 12 h	1 g vancomycin plus 2 g aztreonam per 12 h
Fomin et al. [36]	Na	Multicenter	Double-blind, phase 3	cIAI with bacteremia	Mix	Na	26/23	None	Imipenem-cilastatin	Initial dose of 100 mg, followed by 50 mg every 12 h	500 mg followed by 500 mg per 6 h combined imipenem and cilastatin
Dartois et al. [37]	Na	Multicenter	Double-blind, phase 3	CAP with bacteremia	Mix	Overall	32/31	None	Levofloxacin	Initial dose of 100 mg, followed by 50 mg every 12 h	500 mg per 24 h or per 12 h levofloxacin
Qureshi et al. [38]	2005–09	USA	Retrospective	Bacteremia	KPC-Kp	At 28 days	11/23	Carbapenem aminoglycoside	Carbapenem gentamicin cefepime et al.	Na	Na
Lauf et al. [39]	2006–09	Multicenter	Randomized, double-blind, phase 3	Bacteremia	Mix	Na	7/14	None	Ertapenem ± vancomycin	150 mg once-daily tigecycline	1 g once-daily ertapenem ± vancomycin
Gomez-Simmonds et al. [40]	2006–13	USA	Retrospective	BSI	CR-Kp	At 30 days	26/42	Beta-lactam antibiotic	Polymyxin b aminoglycoside	Initial dose of 100 mg, followed by 50 mg every 12 h	Na
Maki et al. [41]	2007	USA	Prospective	Bacteremia	CVC-CoNS	Na	8/23	Na	Vancomycin	50 mg every 12 h	Na
Oliveira et al. [42]	2009–13	Brazil	Retrospective	Bacteremia	KPC-Enterobacteriaceae	At 30 days	15/62	Carbapenem polymyxin aminoglycoside	Carbapenem polymyxin aminoglycoside	Na	Na

BSI, bloodstream infection; cIAI, complicated intra-abdominal infections; cSSSI, complicated skin and skin-structure infections; CAP, community-acquired pneumonia; CR-Kp, carbapenem-resistant *K. pneumoniae*; CVC-CoNS, central venous catheter-related coagulase-negative staphylococcal; KPC-Kp, *Klebsiella pneumoniae* carbapenemase-producing *K. pneumoniae*; MRSA, methicillin-resistant *Staphylococcus aureus*; Na, not available; VAP, ventilator-associated pneumonia; VRE, vancomycin-resistant enterococci; XDR-Ab, extensively drug-resistant *Acinetobacter baumannii*

### Mortality

**Fig. 1:**
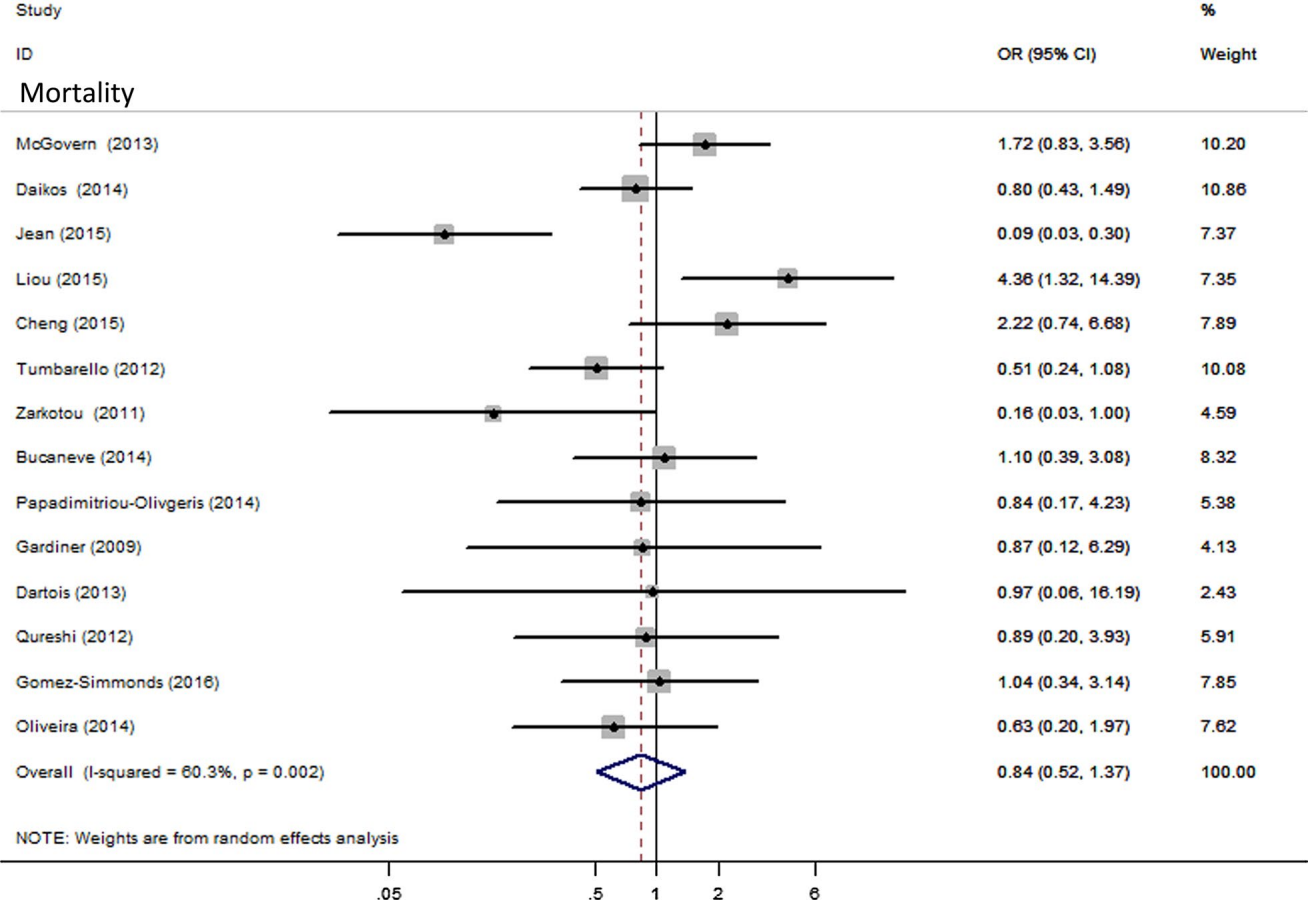
Mortality with tigecycline versus other antibiotics therapy

As shown in Fig. 1, no significant difference was noted when tigecycline was compared with control groups in terms of all-cause mortality (14 studies; 1502 patients) [OR 0.841, 95% confidence interval (CI) 0.517–1.367; P = 0.485]. Because statistical heterogeneity existed among studies (X^2^ = 32.76, df = 13, (P = 0.002), I^2^ = 60.3%), a random-effects model of analysis was used. No publication bias was detected by Egger regression (t = −0.39; df = 12.0; P = 0.701) or Begg (z = 0.55; df = 12.0; P = 0.584).

Table 2 shows the subgroup analysis of the controlled studies. A significant difference was observed between the tigecycline monotherapy therapy group and the tigecycline combination therapy group in terms of mortality (6 studies; 250 patients) (OR 2.733, [95% CI 1.533–4.873; P = 0.001]; I^2^ = 8.7%). A significantly higher mortality was noted in the monotherapy group than in the combination therapy group in cases of blood stream infection. The mortality in the combination of tigecycline plus colistin based group was not significantly lower than that in the other antibiotics combination group (OR 0.68, [95% CI 0.407–1.135; P = 0.14]; I^2^ = 0.0%).

**Table 2:** Subgroup analysis of overall mortality with tigecycline versus other antibiotics for treatment of bloodstram infections in controlled studies

Variables	Studies, no. (patients, no.)	Mortality of tigecycline compared with control OR (95% CI); P	Heterogeneity of studies
Monotherapy vs combination	6 (250)	2.733 (1.533–4.873); 0.001	X^2^ = 5.47, df = 5, (P = 0.361), I^2^ = 8.7%
Tigecycline plus polymyxins based vs other antibiotics combination	5 (289)	0.680 (0.407–1.135); 0.140	X^2^ = 2.88, df = 4, (P = 0.578), I^2^ = 0.0%
Kp BSI	6 (466)	0.678 (0.457–1.006); 0.054	X^2^ = 3.95, df = 5, (P = 0.556), I^2^ = 0.0%
KPC-Kp BSI	5 (398)	0.636 (0.417–0.971); 0.036	X^2^ = 3.31, df = 4, (P = 0.507), I^2^ = 0.0%
Acinetobacter BSI	3 (221)	0.967 (0.096–9.759); 0.978	X^2^ = 23.76, df = 2, (P = 0.001), I^2^ = 91.6%

CI, confidence interval; OR, odds ratio

In the patients infected with *Klebsiella pneumoniae* (Kp) BSI, tigecycline seemed to have a lower mortality than comparator drugs, but the difference was not significant (OR 0.678, [95% CI 0.457–1.006; P = 0.054]; I^2^ = 0.0%; [P = 0.556]). Five studies (398 patients) reported data on carbapenemase-producing Kp BSI, and a significant difference with respect to overall mortality was observed between the tigecycline therapy group and the controls (OR 0.636, [95% CI 0.417–0.971; P = 0.036]; I^2^ = 0.0%; [P = 0.507]). Three controlled studies (221 patients) reported Acinetobacter BSI, no difference was seen between patients who received tigecycline as therapy and others in mortality (OR 0.967, [95% CI 0.096–0.759; P = 0.978]; I^2^ = 91.6%; [P = 0.001]).

### Clinical cure

**Fig. 2:**
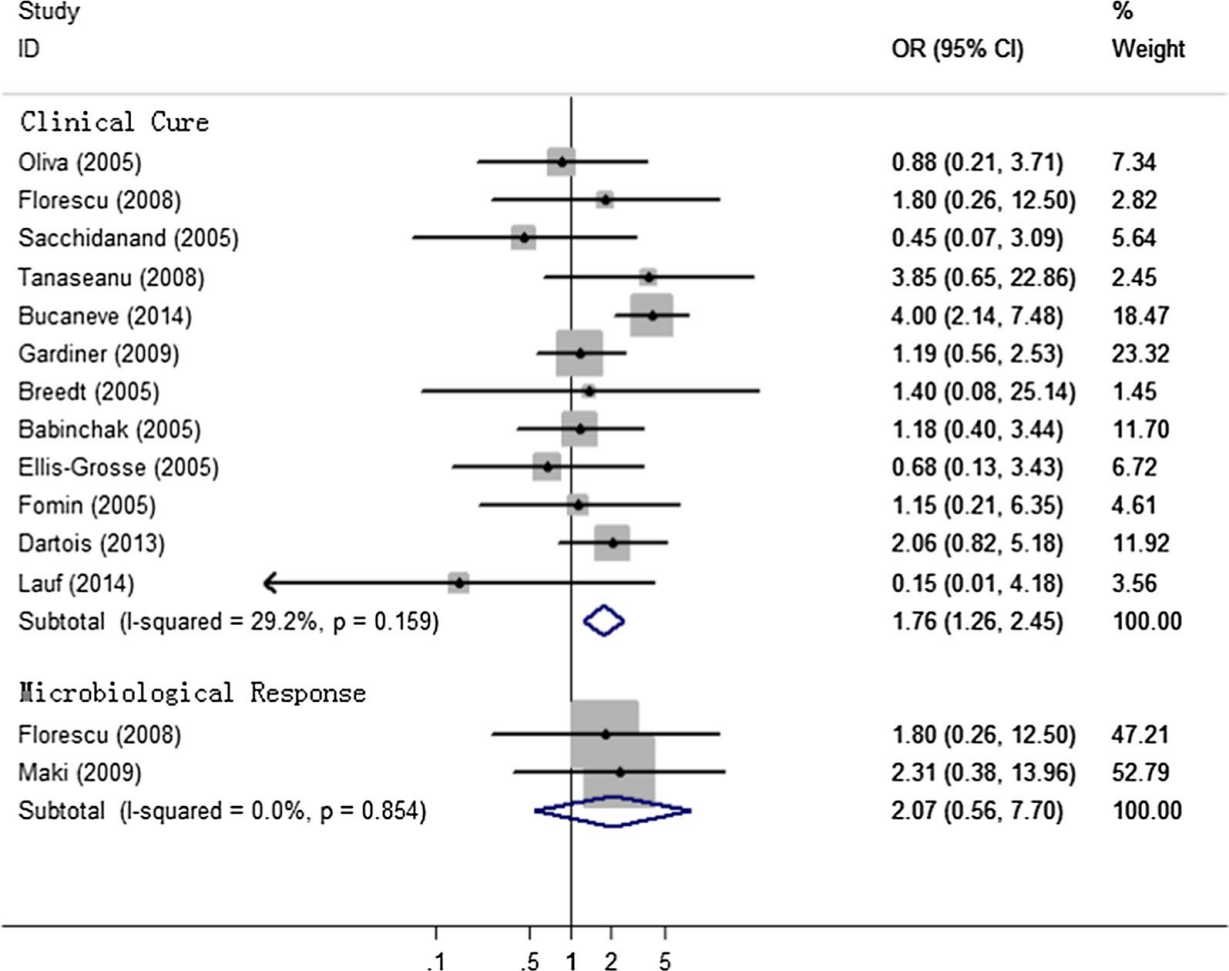
The efficacy of tigecycline, as compared with other antibiotics, in treating infections caused by BSI

There was a significant differences were observed between the tigecycline and control groups in this regard (OR 1.76, [95% CI 1.26–2.45; P = 0.001]; I^2^ = 29.2%; [P = 0.159]; Fig. 2). Clinical cure was significantly higher in the tigecycline population. In the subgroup analysis, for analysis by type of infection, without statistical significance was found in patients with cIAI (OR 0.97, [95% CI 0.52–1.80; P = 0.919]; I^2^ = 0.0%; [P = 0.953]) and cSSSI (OR 0.71, [95% CI 0.26–1.90; P = 0.494]; I^2^ = 0.0%; [P = 0.821]), but in trials assessing patients with CAP, for the rate of clinical cure, the efficacy of tigecycline was better than that of comparator regimens (OR 2.44, [95% CI 1.20–4.94; P = 0.013]; I^2^ = 0.0%; [P = 0.821]). As shown in Fig. 2.

### Microbiological response

As shown in Fig. 2, tigecycline group did not differ significantly compared with the comparators in the rate of microbiological success (OR 2.07, [95% CI 0.56–7.70; P = 0.279]; I^2^ = 0.0%; [P = 0.854]) (Fig. 2).

### Adverse effects

There were not sufficiently effective data to be recoded, so that the common adverse effects of tigecycline (nausea, vomiting, and diarrhea) could not be extracted in any of the studies.

## Discussion

We conducted this systematic review and meta-analysis to investigate the effectiveness and safety of tigecycline for the treatment of BSI. Numerous studies have established bacteremia as a marker of severe infection and a risk for adverse outcomes in multiple treatment settings [9, 10], but there were some positive elements about the treatment of BSI with tigecycline.

To our knowledge, this was the first systematic review to assess the efficacy of tigecycline in treating BSI. Although all-cause mortality was lower with tigecycline than with the control regimens, the difference was not significant. Tigecycline seemed to be better than levofloxacin for treatment of community-acquired pneumonia, and worse than control regimens for cIAI and cSSSI, but these differences were not significant. However, drug safety guidelines published by the FDA refer to an increased mortality risk associated with intravenous tigecycline compared with other drugs used to treat serious infections (risk difference = 0.6%, 95% CI 0.1–1.2) [2]. This result has been confirmed by a study that associated the increased risk mortality with resistant pathogens, hospital-acquired pneumonia, and increased age of patients [11]. However the type of serious infections didn’t include BSI. We used the same effect metric to assess our results, and noted that the risk difference of all-cause mortality was not significant (−3.5%, 95% CI −13 to −6; I^2^ = 85.4%, P = 0.001).

Although the overall mortality did not differ between tigecycline and the control groups, subgroup analysis found the mortality was significantly lower in the tigecycline combination group than in the tigecycline monotherapy therapy group. Tigecycline in combination with colistin, carbapenem in combination with colistin, and tigecycline in combination with gentamicin were the commonly administered antibiotic treatment regimens among the included studies and might result in lower mortality than other combinations of antibiotics. The most common combination was tigecycline with colistin in tigecycline combination therapy group, yet this data did not necessarily predict tigecycline plus polymyxins based therapy was significantly better than other antibiotics combination therapy. For the patients with KPC-Kp BSI, antibiotic therapy with tigecycline was associated with lower mortality.

With regard to clinical response, the evidence that we could compile from studies was that tigecycline therapy may have no clinical advantage over comparator therapy, but may result in better clinical cure in treatment of CAP presenting with bacteremia.

Tigecycline had good eradication ability for most pathogens recorded at baseline, as a novel glycylcycline antibiotic, it has a broad spectrum of antimicrobial activity, ranging from aerobic to anaerobic bacteria, and gram-positive, gram-negative (exceptions of *Pseudomonas aeruginosa* and *Proteus mirabilis*), and atypical organisms [12]. Eradication was better than with control regimens in all cases, although no significant difference was found when tigecycline was compared with the comparators.

Previous studies have shown that the most common adverse effects of tigecycline had increased incidence in the tigecycline group, such as nausea, vomiting, and diarrhea [13, 14]. According to a recently published review, tigecycline induces acute pancreatitis, indicating that surveillance for adverse events from the digestive system is needed during treatment [15]. But lack of data from all trials results can not be obtained about adverse events outcomes in our meta-analysis.

Small non-comparative series have reported relatively poor clinical and microbiological outcomes with tigecycline for tigecycline-susceptible CR-Ab bacteremia [16–18]. The high severity of illness and the notable delays in initiation of effective antimicrobial therapy could also explain these results. In a pooled, retrospective data analysis of phase 3 clinical trials, 91 patients being treated with tigecycline had secondary bacteremia detected, tigecycline appeared safe and well tolerated in the treatment of secondary bacteremia associated with cSSSI, cIAI, and CAP; cure rates were similar to comparative standard therapies [19]. Recently, a high-dose regimen (loading dose 200 mg followed by 100 mg every 12 h) has been successfully and safely used in critically ill patients with severe infections due to multi drug resistant bacteria although the number of primary bacteremia was anecdotal [20].

Several potential limitations should be taken into consideration when interpreting the present results. Firstly, the number of subjects included was not large enough. We would have preferred to contact researchers directly for missing data, but this approach was not attempted because of time constraints. Secondly, in some subgroup analyses, the sample size was small, which may have reduced the power of the statistical analysis. Another important issue is that the administrations of the antibiotics differed among the studies with regard to the duration of infusion or the total daily dose. Thirdly, due to the included studies did not provide relevant data, we were unable to assess the impact of tigecycline on adverse drug reactions. Accordingly, these differences might influence the clinical outcomes. Last, the matter of the emergence of resistance during therapy was not raised by any of the included studies.

In conclusion, based on a review of published cases, tigecycline appears to have produced some favourable clinical and microbiological outcomes in patients with BSI, even when used as monotherapy. This research was needed to clarify whether tigecycline was suitable for treatment such infections when other antibiotics fail, especially because indications for increased risk of all-cause mortality have been reported in patients treated with this drug. The FDA has recently reported an increased risk of death when intravenous tigecycline is used for FDA approved purposes [21], which may be explained by a worsening infection or potential complications [11].

The available evidence suggests that combination antibiotic treatment may offer a comparative advantage over monotherapy with regard to the mortality of critically ill patients with severe infections due to BSI. The number of currently available appropriate antimicrobial agents is limited, combination therapy with tigecycline, it could be a fine option for the treatment of BSI, especially in patients with KPC-Kp BSI.

## Abbreviations

BSI: bloodstream infection
cIAI: complicated intra-abdominal infections
cSSSI: complicated skin and skin-structure infections
CAP: community-acquired pneumonia
CR-Kp: carbapenem-resistant *K. pneumoniae*
CVC-CoNS: central venous catheter-related coagulase-negative staphylococcal
KPC-Kp: *Klebsiella pneumoniae* carbapenemase-producing *K. pneumoniae*
MRSA: methicillin-resistant *Staphylococcus aureus*
VAP: ventilator-associated pneumonia
VRE: vancomycin-resistant enterococci
XDR-Ab: extensively drug-resistant *Acinetobacter baumannii*
